# The relationship between low perceived numeracy and cancer knowledge, beliefs, and affect

**DOI:** 10.1371/journal.pone.0198992

**Published:** 2018-06-11

**Authors:** Katherine Ross, Justin Stoler, Nick Carcioppolo

**Affiliations:** 1 Department of Epidemiology, Rollins School of Public Health, Emory University, Atlanta, Georgia, United States of America; 2 School of Nursing and Health Studies, University of Miami, Coral Gables, Florida, United States of America; 3 Department of Geography and Regional Studies, University of Miami, Coral Gables, Florida, United States of America; 4 Department of Public Health Sciences, Miller School of Medicine, University of Miami, Miami, Florida, United States of America; 5 Department of Communication Studies, University of Miami, Coral Gables, Florida, United States of America; University of Zurich, SWITZERLAND

## Abstract

Low numeracy may skew patient perceptions of information about cancer. This paper examines the relationship between self-reported measures of perceived numeracy and cancer knowledge, beliefs, and affect, using results from 3,052 respondents to the 2007 Health Information National Trends Survey (HINTS-3). Chi-squared tests were used to identify differences in responses between high- and low-numeracy groups using three measures of perceived numeracy. Multivariable logistic regression models were used to evaluate the association between the three perceived numeracy measures and cancer information overload, cancer fatalism, cancer prevention knowledge, and cancer worry. Respondents with low perceived numeracy as expressed by *discomfort* with medical statistics were more likely to report information overload, to display fatalistic attitudes towards cancer, to lack knowledge about cancer prevention, and to indicate that they worried about cancer more frequently. After controlling for sociodemographic characteristics, this measure of perceived numeracy remained significantly associated with information overload, fatalism, lower prevention knowledge, and worry. The other measures of perceived numeracy, which measured *understanding* and *use* of health statistics, were not associated with cancer perceptions. Our findings suggest that individuals with low perceived numeracy broadly differ from individuals with high perceived numeracy in their perceptions of cancer and cancer prevention. By improving our understanding of how perceived numeracy affects patient perceptions of cancer, health providers can improve educational strategies and targeted health messaging.

## Introduction

Health care providers and patients increasingly support shared decision making as the preferred alternative to paternalistic or autonomic approaches to medical decision-making [1]. Patients need the ability to comprehend and utilize health information to meaningfully participate in their own care. This is referred to as “health literacy,” an essential facet of functional adult literacy which shapes personal health outcomes [2–4]. Numeracy is defined as "the ability to access, use, interpret, and communicate mathematical information and ideas, to engage in and manage mathematical demands of a range of situations in adult life” [5]. Health numeracy includes individual skills necessary to understand and use quantitative health information, including reading and interpreting medical information, performing basic computations, and communicating orally about quantitative information [6].

Many Americans score poorly on conventional measures of numeracy. In 2012, the Program for the International Assessment of Adult Competencies (PIACC) conducted a study assessing proficiency in three areas: literacy, numeracy, and problem-solving in a technological environment. While 12% of adults scored on the highest literacy levels, only 9% of Americans scored in the highest numeracy levels. 30% of Americans between the ages of 16 and 65 were at or below level 1, the lowest numeracy proficiency level [5]. Health literacy tends to be particularly low among immigrant populations, racial and ethnic minorities, and low-income populations [7]. A multi-center study of numeracy among Emergency Department patients showed similar demographic disparities [8].

Adequate health numeracy is essential to self-management of several chronic conditions. A study of adults with asthma showed that low numeracy, but not low health literacy alone, was associated with a history of hospitalization for asthma [9]. Low numeracy also mediated disparities in HIV medication adherence among African Americans [10]. Numeracy is also a key factor for effective risk communication. Low numeracy has resulted in distorted risk perceptions and low understanding of screening benefits [11]. This is particularly harmful for chronic disease prevention efforts. While some studies found that numeracy is not correlated with cancer screening uptake [12, 13], others have identified a strong relationship between numeracy and patient ability to accurately determine benefits of mammography [14]. A basic proficiency in numeracy is required to understand online cancer risk information [15] and accurately assess cancer-related health risks [16, 17].

This study evaluates the relationships between three self-reported, perceived low numeracy items and cancer-related knowledge, beliefs, and affect. Some studies have found few differences between subjective (self-reported) and objective (performance-based) measures of health literacy and numeracy [18, 19]. But different relationships between subjective and objective measures of numeracy have been observed with colorectal cancer screening behavior when stratified by perceptions of provider communication [20], and with willingness to pay for breast cancer testing among women in a cancer registry who had higher-risk for carrying the *BRCA1/2* gene [21]. Although the literature acknowledges subjective and objective numeracy as “related but distinct constructs” [22], subjective measures of health numeracy have received less scientific attention even though some, such as Fagerlin et al.’s Subjective Numeracy Scale [23], approximate objective measures, are easier to complete, and are often rated as more acceptable [19, 22].

We also know that holding fatalistic views about cancer, perceiving high cancer information overload, worrying about cancer, and having low cancer knowledge can contribute to the performance of prevention and screening behaviors [24–28]. For example, cancer fatalism, information overload, and low knowledge can all decrease the likelihood of performing cancer screening behaviors; if one believes that cancer cannot be prevented or cured, thinks that news information is conflicting and contradictory, or has a dearth of knowledge about cancer, the enactment of screening behavior is unlikely. Cancer worry is generally positively related to screening behavior; as one tends to worry about the possibility of getting cancer, screening behavior increases [29]. Also, while information overload and prevention knowledge have previously been conceptualized as fatalistic beliefs, more recent measurement research suggests that cancer information overload and cancer fatalism are actually separate constructs [25]. We know much less about the factors underlying the formation of these perceptions. Given the importance of numeracy skills in health information comprehension and decision-making, low perceived numeracy scores may be a crucial individual-level determinant of health disparities by reinforcing cancer-related knowledge, beliefs, and affect which potentially impede timely cancer screening and care.

This study uses results from a large population-based health survey to examine the relationships between three potential proxies for low numeracy and four constructs of cancer perceptions that are associated with the performance of cancer prevention and screening behaviors. Specifically, we test the hypotheses that survey participants perceiving low numeracy will be more likely to express a higher degree of cancer fatalism, cancer information overload, and cancer worry, and express a lower degree of cancer knowledge.

## Materials and methods

### Data

This study uses publicly available data from the third collection of the Health Information and National Trends Survey (HINTS-3), administered by the National Cancer Institute in 2007. To date, HINTS-3 is the only cycle of HINTS in which numeracy was included in the survey. Rationale and methodology for the design of the HINTS survey have been documented in detail elsewhere [30]; the primary purpose was to capture the public’s knowledge, attitudes, and practices related to access, sources, and trust of cancer-related information to help improve health communication. The HINTS survey includes questions about cancer-related health beliefs, sources of health information, current health status, and demographics, and was the first national health survey to include questions about numeracy. This nationally representative survey is conducted both by phone and by mail response cards. Our analysis was restricted to the 4,081 HINTS-3 phone interview respondents with sample weights, as language preference was collected in this mode. Our final analytical sample (*n* = 3,052) excluded 1,029 respondents with incomplete demographic characteristics. A bias analysis of the differences between the included and excluded cases indicates that excluded respondents may have been less diverse than the general population—potentially more likely female and non-white (see S1 Table)—so our analysis of these characteristics can be viewed as a conservative assessment given the incomplete demographics of the excluded cases.

Because the HINTS-3 survey results are a publicly available and anonymized data set (available from the National Cancer Institute at http://hints.cancer.gov), this study was deemed exempt from human subjects review by the University of Miami Institutional Review Board.

### Variables

#### Knowledge, beliefs, and affect

The constructs chosen for the outcomes measures in this study were cancer information overload, cancer fatalism, cancer prevention knowledge, and cancer worry. The respective HINTS-3 questions corresponding to these four constructs were “Agree or disagree: There is so much information out there about cancer that it is hard to know what to do” (information overload), “Agree or disagree: It seems like everything causes cancer” (fatalism), “Agree or disagree: There is not much that you can do to lower your chances of getting cancer” (prevention knowledge), and “How often do you worry about getting cancer?” (worry). Responses to the first three variables were recorded on a four-point Likert scale and stratified into two categories: “strongly agree” and “agree” vs. “strongly disagree” and “disagree.” Available responses to the fourth variable were “never or rarely,” “sometimes,” “often,” or “all the time.” These were collapsed into two categories, “never or rarely” and “sometimes” vs. “often” and”all the time” to provide more comparable sample sizes across categories.

#### Numeracy

There are three survey questions that attempt to measure perceived health numeracy in HINTS-3. The first numeracy-related question (henceforth referred to as “N_understand_”) from the STAT-Confidence Scale [31] asked, “In general, how easy or hard do you find it to understand medical statistics?” The N_understand_ valid responses were “very easy,” “somewhat easy,” “somewhat hard,” and “very hard.” The second question (“N_comfort_”) comes from the Fagerlin Subjective Numeracy Scale [26], and asks, “In general, I am uncomfortable with medical information that uses a lot of statistics.” The N_comfort_ valid responses were “strongly agree,” “agree,” “disagree,” and “strongly disagree.” The third question (“N_use_”) asks, “In general, I use statistics to make decisions about my health.” The N_use_ valid responses were “strongly agree,” “somewhat agree,” “somewhat disagree,” or “strongly disagree.” Responses were recoded into a two-category variable for each of the three numeracy measures: high numeracy (“very easy” and “somewhat easy,” or “strongly agree” and “somewhat agree”) and low numeracy (“somewhat hard” and “very hard,” or “somewhat disagree” and “strongly disagree”). Our modeling approach focused on associations with low perceived numeracy. Note that HINTS-3 did contain a question intended as an objective measure of numeracy: “Which of the following numbers represents the biggest risk of getting a disease?” But this question was only asked in the mail version of the survey, which prevents us from controlling for (and testing potential interactions with) language preference, and assessing N_comfort_, both of which were only asked in the phone survey.

#### Socio-demographic variables

The socio-demographic variables selected for this study are those typically correlated with health literacy [7]: age, gender, race/ethnicity (non-Hispanic White, non-Hispanic Black, Hispanic, non-Hispanic Asian, or other), highest level of education completed (bachelor’s degree or higher, some college, high school diploma, or less than high school), household income (< $25,000, $25,000–34,999, $35,000–49,999, $50,000–74,999, and > $75,000), and preferred language (Spanish or English).

### Statistical analysis

Chi-square tests were used to compare high-numeracy and low-numeracy participant responses to those from the cancer perception questions. All tests were two-sided and were considered statistically significant at the level of α = .05. Binary logistic regression was used to assess the relationship between numeracy and responses to cancer belief questions after adjusting for age, gender, race/ethnicity, income, education, and preferred language. All analyses were conducted in SAS version 9.3 using the HINTS phone interview sample weights to adjust parameter estimates to be representative of the U.S. adult population.

## Results

The demographic characteristics of the 3,052 respondents analyzed from HINTS-3 are presented in Table 1. The weighted study sample was predominantly non-Hispanic white (68.8%) with a slight female majority (50.5%), with 76.7% completing at least a high school diploma, and 25.7% attaining a Bachelor’s degree or higher. Just 31.9% of participants had an annual household income of greater than $75,000, with 53.2% over $50,000. The mean age of the sample was 55.3 years old (standard deviation [SD] = 16.4 years), with a range of 18–97. The three perceived numeracy measures also were associated with many of the demographic characteristics. Respondents with low N_understand_ were more likely to be of ethnic/racial minority status (*X*^2^ = 18.3, *P* < .001), report lower household income (*X*^2^ = 18.3, *P* < .001), have lower educational attainment (*X*^2^ = 58.3, *P* < .001), and be more likely to prefer Spanish (*X*^2^ = 9.9, *P* = .002). Respondents with low N_comfort_ were more likely to be female (*X*^2^ = 10.0, *P* = .002), be of ethnic/racial minority status (*X*^2^ = 11.1, *P* = .010), report lower household income (*X*^2^ = 49.4, *P* < .001), have lower educational attainment (*X*^2^ = 49.4, *P* < .001), and be more likely to prefer Spanish (*X*^2^ = 9.3, *P* = .002). Respondents with low N_use_ were more likely to report lower household income (*X*^2^ = 10.4, *P* = .030), and have lower educational attainment (*X*^2^ = 10.2, *P* = .020). These results underscore the relationships between socio-demographic characteristics and perceived numeracy and the importance of controlling for these characteristics in a multi-variable modeling approach.

**Table 1:** Demographic characteristics (as weighted percentages) of 3,052 phone respondents analyzed from HINTS-3, and by perceived numeracy measure. Italics denote statistical significance of chi-square test at *P* < .05.

Characteristic	All	N_understand_		N_comfort_		N_use_	
		Low	High	Low	High	Low	High
Gender							
Male	49.5	48.8	51.2	*45*.*8*	*53*.*9*	46.1	51.9
Female	50.5	53.5	48.3	*54*.*2*	*46*.*1*	54.9	48.1
Race/Ethnicity							
Non-Hispanic White	68.8	*61*.*8*	*72*.*7*	*64*.*9*	*73*.*7*	68.7	69.1
Hispanic	13.6	*11*.*0*	*10*.*5*	*16*.*4*	*10*.*4*	13.0	14.0
Non-Hispanic Black	11.7	*12*.*0*	*11*.*7*	*12*.*5*	*10*.*6*	14.2	10.3
Other	5.8	*7*.*2*	*5*.*1*	*6*.*2*	*5*.*3*	4.2	6.7
Household Income							
< $20,000	16.0	*21*.*8*	*12*.*0*	*21*.*1*	*9*.*6*	*17*.*1*	*15*.*0*
$20,000 to $34,999	17.3	*20*.*3*	*15*.*4*	*18*.*9*	*15*.*3*	*20*.*1*	*15*.*4*
$35,000 to $49,999	13.6	*15*.*0*	*12*.*7*	*15*.*8*	*11*.*0*	*11*.*7*	*14*.*7*
$50,000 to $74,999	21.3	*20*.*2*	*22*.*1*	*20*.*7*	*22*.*1*	*22*.*1*	*21*.*0*
> $75,000	31.9	*22*.*7*	*37*.*7*	*23*.*5*	*41*.*9*	*29*.*0*	*33*.*9*
Highest Education Completed							
Less than High School	13.3	*21*.*4*	*8*.*3*	*15*.*6*	*10*.*4*	*15*.*1*	*11*.*7*
High School Diploma	30.0	*33*.*9*	*27*.*8*	*35*.*6*	*23*.*5*	*30*.*9*	*29*.*5*
Some College	21.0	*26*.*4*	*33*.*8*	*30*.*4*	*31*.*7*	*33*.*3*	*29*.*8*
College Degree or higher	25.7	*18*.*3*	*30*.*2*	*18*.*4*	*34*.*4*	*20*.*7*	*29*.*0*
Preferred Language							
English	92.0	*88*.*4*	*94*.*0*	*89*.*1*	*95*.*3*	91.9	92.1
Spanish	8.0	*11*.*6*	*6*.*0*	*10*.*9*	*4*.*7*	8.1	7.9

Regarding perceived numeracy, 1,162 participants (38.6%) answered “hard” or “very hard” to the first numeracy-related question, “In general, how easy or how hard do you find it to understand medical statistics?” These participants were classified as *low N*_*understand*_. 1,679 participants (55.5%) strongly agreed or agreed with the second numeracy-related statement, “In general, I am uncomfortable with medical information that uses a lot of statistics.” These participants were classified as *low N*_*comfort*_. 1,143 participants (37.8%) indicated that they disagreed or strongly disagreed with the third numeracy-related statement, “In general, I use statistics to make decisions about my health.” These participants were classified as *low N*_*use*_.

Participants with low N_understand_ and participants with low N_comfort_ had similar response patterns to the first three belief questions (Table 2). Participants with low perceived numeracy (N_understand_ or N_comfort_) were significantly more likely than their higher-numeracy counterparts to answer the cancer belief questions in a way that demonstrates cancer information overload (N_understand_: *X*^2^ = 20.5, *P* < .001; N_comfort_: *X*^*2*^ *=* 60.8, *P* < .001), express fatalistic beliefs about cancer (N_understand_: *X*^2^ = 4.5, *P* = 0.03; N_comfort_: *X*^*2*^ = 15.3, *P* < .001), demonstrate low cancer prevention knowledge (N_understand_: *X*^*2*^ = 21.9, *P* < .001; N_comfort_: *X*^*2*^ *=* 53.4, *P* < .001), and express cancer worry (i.e. about getting cancer “often” or “all the time”) (N_understand_: *X*^*2*^ = 9.1, *P* = 0.003; N_comfort_: *X*^*2*^ *=* 23.1, *P* < .001). There were no statistically significant differences in the rates of the four cancer belief responses between persons with low N_use_ and high N_use_.

**Table 2:** Bivariate associations of each belief outcome measure with each of the three perceived numeracy measures^a^.

Characteristic	% of respondents					
	N_understand_		N_comfort_		N_use_	
	High	Low	High	Low	High	Low
Difficult to Understand Stats (low N_understand_)	--	--	21.8	50.8***	31.8	46.0***
Uncomfortable with Stats (low N_comfort_)	42.4	73.1***	--	--	54.3	53.0
Do Not Use Stats for Decisions (low N_use_)	32.6	46.8***	38.6	37.4	--	--
Information Overload (High)	74.1	83.7***	68.9	85.3***	77.1	78.5
Fatalism (High)	47.1	53.6*	43.2	55.0***	50.9	47.7
Prevention Knowledge (Low)	22.4	33.4***	17.5	34.3***	28.1	24.3
Frequency of Worry (High)	6.7	10.6**	5.4	10.4***	8.0	8.3

^a^ Table should be interpreted by starting with a column, i.e. for respondents whose N_understand_ was *High*, 74.1% reported High Information Overload.

* *p*<0.05;

** *p*<0.01;

*** *p*<0.001; significance from *X*^2^ test

After controlling for age, race, gender, education, preferred language, household income, and the other low perceived numeracy measures (Table 3), low N_comfort_ remained significantly associated with fatalism (OR 1.63, 95% CI 1.23–2.14, *P* < .001), information overload (OR 2.37, 95% CI 1.79–3.13, *P* < .001), low prevention knowledge (OR 1.79, 95% CI 1.32–2.42, *P* < .001), and high frequency of worry (OR 1.68, 95% CI 1.14–2.49, P = 0.01). Low N_understand_ had a modest but statistically significant association with information overload (OR 1.39, 95% CI 1.04–1.87, *P* = 0.03), but not with fatalism (OR 1.22, 95% CI 0.95–1.59, P = 0.12), prevention knowledge (OR 1.11, 95% CI 0.82–1.50, P = 0.49) or high frequency of worry (OR 1.30, 95% CI 0.95–1.80, *P* = 0.59). Low N_use_ was neither significantly associated with any of the four belief questions after adjusting for other factors, nor did the presence of N_use_ affect the influence of N_understand_ or N_comfort_ in any of the models. Each numeracy measure was originally tested independently (i.e. without the presence of the other two numeracy measures) in multivariable models adjusting for sociodemographic covariates, and the results were nearly identical to those presented in the full model in Table 3 (refer to S2–S9 Tables for full results). In addition, we tested all six combinations of two- and three-way interactions between the perceived numeracy measures, and none of the interaction terms were statistically significant.

**Table 3:** Multivariable logistic regression models of fatalism, information overload, prevention knowledge, and worry on three measures of low numeracy (N_understand_, N_comfort_, and N_use_) adjusted for sex, age, race/ethnicity, education, household income, and preferred language.

Characteristic	Fatalism	Information Overload	Prevention Knowledge	Worry
	OR (95% CI)	OR (95% CI)	OR (95% CI)	OR (95% CI)
Low Numeracy (N_understand_)	1.22 (0.95–1.59)	1.39* (1.04–1.87)	1.11 (0.2–1.50)	1.30 (0.95–1.80)
Low Numeracy (N_comfort_)	1.63***(1.23–2.14)	2.37***(1.79–3.13)	1.79***(1.32–2.42)	1.68*(1.14–2.49)
Low Numeracy (N_use_)	0.84 (0.67–1.06)	1.03 (0.75–1.41)	0.75 (0.54–1.05)	1.00 (0.66–1.53)
Household Income				
> $75,000 †				
$50,000–$75,000	0.87 (0.64–1.18)	1.13 (0.79–1.62)	1.06 (0.70–1.67)	1.20 (0.73–1.98)
$35,000–$50,000	0.99 (0.70–1.41)	1.09 (0.72–1.65)	1.27 (0.73–2.19)	1.19 (0.61–2.34)
$20,000–$35,000	1.12 (0.76–1.66)	1.34 (0.85–2.10)	1.56 (0.98–2.50)	1.53 (0.81–2.91)
< $20,000	0.99 (0.67–1.45)	1.30 (0.82–2.06)	2.08** (1.30–3.34)	1.16 (0.65–2.07)
Race/Ethnicity				
Non-Hispanic White †				
Hispanic	0.70 (0.40–1.23)	0.90 (0.51–1.58)	1.82* (1.04–3.18)	0.88 (0.37–2.13)
Black	0.71 (0.43–1.16)	0.76 (0.46–1.27)	0.90 (0.56–1.45)	0.22**(0.09–0.57)
Other	0.67 (0.39–1.16)	0.84 (0.44–1.58)	2.48** (1.38–4.44)	1.75 (0.74–4.15)
Male	0.91 (0.71–1.16)	1.26 (0.95–1.67)	1.08 (0.80–1.45)	0.66 (0.40–1.11)
Age (years)	0.99** (0.98–0.99)	0.99* (0.98–0.99)	1.01 (0.99–1.02)	0.99 (0.98–1.01)
Preferred Language: Spanish	0.60 (0.27–1.30)	0.70 (0.31–1.59)	1.70 (0.78–3.71)	2.78* (1.08–7.16)
Education				
Bachelors or Higher †				
Some College	1.51**((1.15–1.99)	1.91**(1.30–2.79)	1.70*(1.08–2.65)	1.09 (0.72–1.65)
High School	1.55**(1.17–2.06)	1.67*(1.13–2.48)	2.22***(1.59–3.08)	1.19 (0.74–1.92)
Less than High School	1.56* (1.02–2.38)	1.52 (0.70–3.30)	3.41***(2.04–5.69)	1.15 (0.57–2.34)

^†^ Reference category;

* *p*<0.05;

** *p*<0.01;

*** *p*<0.001

## Discussion

This study examined the association between low perceived numeracy and responses to questions concerning cancer-related knowledge, beliefs, and affect in the 2007 HINTS-3 survey. Chi-square tests showed statistically significant differences between high- and low-numeracy group responses for N_understand_ and N_comfort_ concerning information overload, fatalism, prevention knowledge, and worry about cancer. After controlling for sociodemographic factors, N_comfort_ continued to have a significant positive association with information overload, fatalism, and worry, and a significant negative association with prevention knowledge. This is notable in light of the strong significant association between lower levels of education and these three constructs.

Only one of the three perceived numeracy measures was associated with cancer knowledge, beliefs and affect: respondents who were “uncomfortable with medical information that uses a lot of statistics” (N_comfort_). This response is, at face value, a tacit acknowledgment of limited skills by the survey respondent. Our analysis does not support using the other perceived numeracy measures, N_understand_ and N_use_, for predicting cancer perceptions. This is likely because phrasing of the original questions does not necessarily capture the degree of confidence in one’s understanding or use of statistics in decision-making. Respondents agreeing with these questions—whom in this study were modeled as high numeracy—may not understand the statistics they are using (and conversely those who do not use statistics may still understand them), which renders these questions a poor proxy for numeracy. It is also possible that the potential bias introduced via case exclusion due to missing demographic information is obscuring some of the relationships tested, particularly the lack of significant associations for N_understand_ and N_use_, which exhibit lower levels of low perceived numeracy in the excluded cases.

Many public health campaigns utilize statistics as a part of persuasive message design. For low numeracy individuals that lack the ability to correctly interpret these statistics, messages could be distorted or lost. Notably, 68.9% of the high N_comfort_ group and 85.3% of the low N_comfort_ group reported feeling overwhelmed by the amount of recommendations about cancer prevention, and approximately half of each group (43.2% vs. 55% respectively) agreed with the statement “it seems like everything causes cancer.” These findings point to a need for more cohesive and purposefully framed health education messages that account for the relatively low numeracy of the general population. In particular, the current findings highlight the possible unintended effects of message design when incorporating complicated numerical risk information into cancer prevention and screening messages for use among low-numeracy audiences. It is possible that current messaging strategies designed to increase perceived susceptibility and severity of cancer risk may contribute to the formation of detrimental beliefs and affect that may reduce the likelihood of performing cancer prevention and screening behaviors. Given this consideration, it is of critical importance for future research to better understand whether the conveyance of numerical risk information can yield unintended outcomes among a low-numeracy audience.

Additionally, numeracy likely affects depth of cancer news and intervention message processing. Fuzzy trace theory [32] describes how people process risk information, specifying a dual process model of gist and verbatim understanding. Verbatim understanding involves the ability to recite statistics or percentages, whereas gist understanding involves a qualitative conceptualization of risk that represents the underlying meaning of a statistic or percentage. Decision-making is based predominantly on gist interpretations of statistics. Previous work on fuzzy trace theory suggests that numeracy skills affect the formation of accurate gist understanding from verbatim recollection of statistics [33]. It thus appears critical that numeracy be included in future cancer prevention and screening interventions to better predict proximal outcomes of message exposure, such as cancer information overload, and even more distal outcomes including intentions and behavior.

Our analysis of the HINTS-3 data set is limited by the survey’s single-item measures of perceived numeracy that relied on unvalidated, self-reported evaluations, though our regression models of these single-item measures are robust across multiple cancer perception constructs. We view the results are compelling given the parsimony of these single-item measures, and this analysis beckons a comparison of these relationships with cancer perceptions to more nuanced measures of subjective and objective numeracy. Future comparisons might employ variable reduction techniques (e.g., principle components analysis) to test whether multiple single items could be synthesized into a single numeracy construct, and perhaps incorporate the lone objective numeracy item that was only asked in the HINTS mail sample. While our models control for known confounders, there likely are additional factors that mediate the relationship between numeracy and cancer perceptions. In addition, this data is from 2007; while more recent comparison data remains unavailable, perceptions such as *worry* have long been understood to be related to trait anxiety [34], as opposed to being an ephemeral emotion, thus underscoring the ongoing relevance of these 2007 HINTS items.

Future studies should focus on how numeracy affects cancer-related knowledge, beliefs, and affect, as our cross-sectional study design does not allow us to infer causality between numeracy and cancer beliefs. One topic in need of further study is the relationship between numeracy, information seeking, and knowledge about cancer. Higher numeracy has been associated with more positive information-seeking experiences [35], and this association may mediate the relationship between numeracy and perceptions about cancer.

Regardless, this study highlights the robust association of a simple perceived numeracy measure on cancer perceptions, and presents an opportunity to develop and test health promotion messages that are tailored to individuals with low numeracy given the evidence that fatalism [26, 27, 36], information overload [25], and low prevention knowledge [13] can all hinder health promotion efforts. Low numeracy was not as strongly associated with increased cancer worry, which is consistent with studies observing that cancer worry can facilitate, rather than inhibit, screening [24], and can be alleviated by increases in perceived cancer knowledge [28]. This type of tailored messaging offers a potential vehicle for improving shared cancer decision-making between patients and clinicians, and may have broader implications for other types of chronic disease management.

## Conclusion

This study explored the relationship between perceived numeracy and cancer-related perceptions measured by HINTS. Our findings suggest that individuals with low perceived numeracy differ from individuals with high perceived numeracy in perceptions of cancer and cancer prevention, which may increase avoidance of cancer prevention and screening behaviors. As the volume of quantitative information and health data available to consumers continues to rise, particularly through new channels such as mobile and wearable technologies, it becomes essential to understand how consumers interpret and utilize this information. This study has implications for public health professionals, health communication specialists, and health care providers interested in the production and effectiveness of targeted health messages given the natural population variation in numeracy. Future research should extend this work to explore the potential associations between low numeracy, health behaviors, and clinical outcomes.

## Supporting information

S1 Table Bias analysis to assess socio-demographic differences between HINTS-3 cases included (n = 3,052) and excluded (n = 1,029) from analysis. (DOCX)

S2 Table Adjusted multivariable logistic regression models of the relationship between each of the three measures of numeracy (N_understand_, N_comfort_, and N_use_) and information overload, controlling for select sociodemographic characteristics. (DOCX)

S3 Table Adjusted multivariable logistic regression models of the relationship between each of the three measures of numeracy (N_understand_, N_comfort_, and N_use_) and fatalism, controlling for select sociodemographic characteristics. (DOCX)

S4 Table Adjusted multivariable logistic regression models of the relationship between each of the three measures of numeracy (N_understand_, N_comfort_, and N_use_) and prevention knowledge, controlling for select sociodemographic characteristics. (DOCX)

S5 Table Adjusted multivariable logistic regression models of the relationship between each of the three measures of numeracy (N_understand_, N_comfort_, and N_use_) and worry, controlling for select sociodemographic characteristics. (DOCX)

S6 Table Adjusted multivariable logistic regression models of the relationship between each of the three measures of numeracy (N_understand_, N_comfort_, and N_use_) and information overload, controlling for select sociodemographic characteristics and additional numeracy measures. (DOCX)

S7 Table Adjusted multivariable logistic regression models of the relationship between each of the three measures of numeracy (N_understand_, N_comfort_, and N_use_) and fatalism, controlling for select sociodemographic characteristics and additional numeracy measures. (DOCX)

S8 Table Adjusted multivariable logistic regression models of the relationship between each of the three measures of numeracy (N_understand_, N_comfort_, and N_use_) and prevention knowledge, controlling for select sociodemographic characteristics and additional numeracy measures. (DOCX)

S9 Table Adjusted multivariable logistic regression models of the relationship between each of the three measures of numeracy (N_understand_, N_comfort_, and N_use_) and worry, controlling for select sociodemographic characteristics and additional numeracy measures. (DOCX)

## References

[1] Kon AA. The shared decision-making continuum. JAMA. 2010;304(8):903–4. doi: 10.1001/jama.2010.1208 20736477 10.1001/jama.2010.1208

[2] Berkman ND, Sheridan SL, Donahue KE, Halpern DJ, Crotty K. Low health literacy and health outcomes: an updated systematic review. Annals of Internal Medicine. 2011;155(2):97–107. doi: 10.7326/0003-4819-155-2-201107190-00005 21768583 10.7326/0003-4819-155-2-201107190-00005

[3] Institute of Medicine. Health literacy: a prescription to end confusion. Washington, DC: National Academies Press; 2004. 25009856

[4] Nutbeam D. The evolving concept of health literacy. Social Science & Medicine. 2008;67(12):2072–8. https://doi.org/10.1016/j.socscimed.2008.09.050. 18952344 10.1016/j.socscimed.2008.09.050

[5] Centers for Disease Control and Prevention. Understanding literacy and numeracy 2014 [cited 2015 3 March]. http://www.cdc.gov/healthliteracy/learn/understandingliteracy.html.

[6] Ancker JS, Kaufman D. Rethinking health numeracy: A multidisciplinary literature review. JAMIA. 2007;14(6):713–21. doi: 10.1197/jamia.M2464 17712082 10.1197/jamia.M2464 PMC2213486

[7] Kutner M, Peters, E., Jin, Y., and Paulsen, C. Health literacy of America’s adults: Results from the 2003 National Assessment of Adult Literacy. National Center for Education, Statistics, 2006 Contract No.: Report.

[8] Ginde AA, Clark S, Goldstein JN, Camargo CA. Demographic disparities in numeracy among emergency department patients: Evidence from two multicenter studies. Patient Education and Counseling. 2008;72(2):350–6. doi: 10.1016/j.pec.2008.03.012 18462915 10.1016/j.pec.2008.03.012

[9] Apter AJ, Cheng J, Small D, Bennett IM, Albert C, Fein DG, et al Asthma numeracy skill and health literacy. Journal of Asthma. 2006;43(9):705–10. doi: 10.1080/02770900600925585 17092853 10.1080/02770900600925585

[10] Waldrop-Valverde D, Osborn CY, Rodriguez A, Rothman RL, Kumar M, Jones DL. Numeracy skills explain racial differences in HIV medication management. AIDS and Behavior. 2010;14(4):799–806. doi: 10.1007/s10461-009-9604-4 19669403 10.1007/s10461-009-9604-4 PMC2891293

[11] Reyna VF, Nelson WL, Han PK, Dieckmann NF. How numeracy influences risk comprehension and medical decision making. Psychological Bulletin. 2009;135(6):943–73. doi: 10.1037/a0017327 19883143 10.1037/a0017327 PMC2844786

[12] Aggarwal A, Speckman JL, Paasche-Orlow MK, Roloff KS, Battaglia TA. The role of numeracy on cancer screening among urban women. American Journal of Health Behavior. 2007;31 Suppl 1:S57–S68. 17931137 10.5555/ajhb.2007.31.supp.S57

[13] Schapira MM, Neuner J, Fletcher KE, Gilligan MA, Hayes E, Laud P. The relationship of health numeracy to cancer screening. Journal of Cancer Education. 2011;26(1):103–10. doi: 10.1007/s13187-010-0133-7 20577913 10.1007/s13187-010-0133-7 PMC4162638

[14] Schwartz LM, Woloshin S, Black WC, Welch HG. The role of numeracy in understanding the benefit of screening mammography. Annals of Internal Medicine. 1997;127(11):966–72. 9412301 10.7326/0003-4819-127-11-199712010-00003

[15] Donelle L, Arocha JF, Hoffman-Goetz L. Health literacy and numeracy: Key factors in cancer risk comprehension. Chronic Diseases in Canada. 2008;29(1):1–8. 19036218

[16] Davids SL, Schapira MM, McAuliffe TL, Nattinger AB. Predictors of pessimistic breast cancer risk perceptions in a primary care population. Journal of General Internal Medicine. 2004;19(4):310–5. Epub 2004/04/06. doi: 10.1111/j.1525-1497.2004.20801.x . 15061739 10.1111/j.1525-1497.2004.20801.x PMC1492192

[17] Weinstein ND, Atwood K, Puleo E, Fletcher R, Colditz G, Emmons K. Colon cancer: risk perceptions and risk communication. Journal of Health Communication. 2004;9(1):53–65. Epub 2004/02/06. doi: 10.1080/10810730490271647 . 14761833 10.1080/10810730490271647

[18] Kiechle ES, Bailey SC, Hedlund LA, Viera AJ, Sheridan SL. Different measures, different outcomes? A systematic review of performance-based versus self-reported measures of health literacy and numeracy. Journal of General Internal Medicine. 2015;30(10):1538–46. Epub 2015/04/29. doi: 10.1007/s11606-015-3288-4 . 25917656 10.1007/s11606-015-3288-4 PMC4579206

[19] Zikmund-Fisher BJ, Smith DM, Ubel PA, Fagerlin A. Validation of the Subjective Numeracy Scale: Effects of low numeracy on comprehension of risk communications and utility elicitations. Medical Decision Making. 2007;27(5):663–71. doi: 10.1177/0272989X07303824 . 17652180 10.1177/0272989X07303824

[20] Ciampa PJ, Osborn CY, Peterson NB, Rothman RL. Patient numeracy, perceptions of provider communication, and colorectal cancer screening utilization. Journal of Health Communication. 2010;15(sup3):157–68. doi: 10.1080/10810730.2010.522699 21154091 10.1080/10810730.2010.522699 PMC3075203

[21] Miron-Shatz T, Hanoch Y, Katz BA, Doniger GM, Ozanne EM. Willingness to test for BRCA1/2 in high risk women: Influenced by risk perception and family experience, rather than by objective or subjective numeracy? Judgment and Decision Making. 2015;10(4):386–99.

[22] Dolan JG, Cherkasky OA, Li Q, Chin N, Veazie PJ. Should health numeracy be assessed objectively or subjectively? Medical Decision Making. 2016;36(7):868–75. doi: 10.1177/0272989X15584332 . 25948493 10.1177/0272989X15584332 PMC4636483

[23] Fagerlin A, Zikmund-Fisher BJ, Ubel PA, Jankovic A, Derry HA, Smith DM. Measuring Numeracy without a Math Test: Development of the Subjective Numeracy Scale. Medical Decision Making. 2007;27(5):672–80. doi: 10.1177/0272989X07304449 . 17641137 10.1177/0272989X07304449

[24] Hay JL, Buckley TR, Ostroff JS. The role of cancer worry in cancer screening: A theoretical and empirical review of the literature. Psycho-Oncology. 2005;14(7):517–34. doi: 10.1002/pon.864 15490428 10.1002/pon.864

[25] Jensen JD, Carcioppolo N, King AJ, Scherr CL, Jones CL, Niederdieppe J. The cancer information overload (CIO) scale: Establishing predictive and discriminant validity. Patient Education and Counseling. 2014;94(1):90–6. doi: 10.1016/j.pec.2013.09.016 24268921 10.1016/j.pec.2013.09.016

[26] Niederdeppe J, Levy AG. Fatalistic beliefs about cancer prevention and three prevention behaviors. Cancer Epidemiology, Biomarkers and Prevention. 2007;16(5):998–1003. Epub 2007/05/18. doi: 10.1158/1055-9965.EPI-06-0608 . 17507628 10.1158/1055-9965.EPI-06-0608

[27] Powe BD, Finnie R. Cancer fatalism: The state of the science. Cancer Nursing. 2003;26(6):454–67. 15022977 10.1097/00002820-200312000-00005

[28] Lee SY, Hawkins RP. Worry as an uncertainty-associated emotion: exploring the role of worry in health information seeking. Health Communication. 2016;31(8):926–33. doi: 10.1080/10410236.2015.1018701 26752071 10.1080/10410236.2015.1018701

[29] Jensen JD, Bernat JK, Davis LA, Yale R. Dispositional cancer worry: convergent, divergent, and predictive validity of existing scales. Journal of Psychosocial Oncology. 2010;28(5):470–89. doi: 10.1080/07347332.2010.498459 20730660 10.1080/07347332.2010.498459 PMC2928149

[30] Nelson D, Kreps G, Hesse B, Croyle R, Willis G, Arora N, et al The Health Information National Trends Survey (HINTS): Development, design, and dissemination. Journal of Health Communication. 2004;9(5):443–60. doi: 10.1080/10810730490504233 15513791 10.1080/10810730490504233

[31] Woloshin S, Schwartz LM, Welch HG. Patients and medical statistics: Interest, confidence, and ability. Journal of General Internal Medicine. 2005;20(11):996–1000. doi: 10.1111/j.1525-1497.2005.00179.x 16307623 10.1111/j.1525-1497.2005.00179.x PMC1490265

[32] Reyna VF. A theory of medical decision making and health: Fuzzy trace theory. Medical Decision Making. 2008;28(6):850–65. doi: 10.1177/0272989X08327066 19015287 10.1177/0272989X08327066 PMC2617718

[33] Reyna VF, Brainerd CJ. The importance of mathematics in health and human judgment: Numeracy, risk communication, and medical decision making. Learning and Individual Differences. 2007;17(2):147–59. http://dx.doi.org/10.1016/j.lindif.2007.03.010.

[34] Mathews A. Why worry? The cognitive function of anxiety. Behaviour Research and Therapy. 1990;28(6):455–68. http://dx.doi.org/10.1016/0005-7967(90)90132-3. 2076083 10.1016/0005-7967(90)90132-3

[35] Chen YX, Feeley TH. Numeracy, information seeking, and self-efficacy in managing health: An analysis using the 2007 Health Information National Trends Survey (HINTS). Health Communication. 2014;29(9):843–53. doi: 10.1080/10410236.2013.807904 24266723 10.1080/10410236.2013.807904

[36] Kobayashi LC, Smith SG. Cancer fatalism, literacy, and cancer information seeking in the American public. Health Education and Behavior. 2015 Epub 2015/09/18. doi: 10.1177/1090198115604616 . 26377524 10.1177/1090198115604616 PMC5123630

