# Supplementary material for: The relationship between low perceived numeracy and cancer knowledge, beliefs, and affect
Source: PLoS One. 2018 Jun 11;13(6):e0198992. doi: 10.1371/journal.pone.0198992 (PMC5995386; doi:10.1371/journal.pone.0198992)
Supplement: S1 Table — (DOCX) [file pone.0198992.s001.docx]

The Relationship between Low Perceived Numeracy and Cancer Knowledge, Beliefs, and Affect

S1 Table. Bias analysis to assess socio-demographic differences between HINTS-3 cases included (*n* = 3,052) and excluded (*n* = 1,029) from analysis.

| Characteristic | Included | | Excluded | | *Χ*^2^ | *P*-value |
| --- | --- | --- | --- | --- | --- | --- |
|  | *n* | % | *n* | % |  |  |
| Gender |  |  |  |  | 9.4 | 0.002 |
| Male | 1226 | 40.2 | 358 | 34.8 |  |  |
| Female | 1826 | 59.8 | 671 | 65.2 |  |  |
| Race/Ethnicity |  |  |  |  | 612.0 | <0.001 |
| Non-Hispanic White | 2421 | 79.3 | 545 | 56.2 |  |  |
| Hispanic | 258 | 8.5 | 40 | 4.1 |  |  |
| Non-Hispanic Black | 208 | 6.8 | 39 | 4 |  |  |
| Other | 165 | 5.4 | 346 | 35.7 |  |  |
| Household Income |  |  |  |  | 6.8 | 0.14 |
| < $20,000 | 514 | 16.8 | 12 | 29.2 |  |  |
| $20,000 to $34,999 | 558 | 18.3 | 8 | 19.5 |  |  |
| $35,000 to $49,999 | 397 | 13 | 7 | 17.1 |  |  |
| $50,000 to $74,999 | 579 | 19 | 6 | 14.6 |  |  |
| > $75,000 | 1004 | 32.9 | 8 | 19.5 |  |  |
| Highest Education Completed |  |  |  |  | 6.2 | 0.10 |
| Less than High School | 287 | 9.4 | 80 | 11.4 |  |  |
| High School Diploma | 784 | 25.7 | 201 | 28.6 |  |  |
| Some College | 860 | 28.2 | 187 | 26.6 |  |  |
| College Degree or higher | 1122 | 36.7 | 236 | 33.5 |  |  |
| Preferred Language |  |  |  |  | 3.7 | 0.06 |
| English | 2924 | 95.8 | 971 | 94.4 |  |  |
| Spanish | 128 | 4.2 | 58 | 5.4 |  |  |
| Numeracy |  |  |  |  |  |  |
| Difficult to Understand Stats (low N_understand_) | 1162 | 38.6 | 312 | 43.2 | 5.1 | 0.02 |
| Uncomfortable with Stats (low N_comfort_) | 1679 | 55.5 | 418 | 57.8 | 1.2 | 0.26 |
| Do Not Use Stats for Decisions (low N_use_) | 1143 | 37.8 | 307 | 42.5 | 5.4 | 0.02 |
