# Supplementary material for: The relationship between low perceived numeracy and cancer knowledge, beliefs, and affect
Source: PLoS One. 2018 Jun 11;13(6):e0198992. doi: 10.1371/journal.pone.0198992 (PMC5995386; doi:10.1371/journal.pone.0198992)
Supplement: S5 Table — (DOCX) [file pone.0198992.s005.docx]

The Relationship between Low Perceived Numeracy and Cancer Knowledge, Beliefs, and Affect

S5 Table. Adjusted multivariable logistic regression models of the relationship between each of the three measures of numeracy (N_understand_, N_comfort_, and N_use_) and worry, controlling for select sociodemographic characteristics.

|  | N_understand_ | |  | N_comfort_ | |  | N_use_ | |
| --- | --- | --- | --- | --- | --- | --- | --- | --- |
| Characteristic | *β* (SE) | OR (95% CI) |  | *β* (SE) | OR (95% CI) |  | *β* (SE) | OR (95% CI) |
| Constant | -2.61 (0.32) |  |  | -2.75 (0.32) |  |  | -2.62 (0.33) |  |
| Low Numeracy | 0.37 (0.16) | 1.44*(1.05, 1.99) |  | 0.58 (0.19) | 1.79**(1.22, 2.65) |  | 0.02 (0.21) | 1.02 (0.67, 1.55) |
| Household Income |  |  |  |  |  |  |  |  |
| > $75,000 † |  |  |  |  |  |  |  |  |
| $50,000 - $75,000 | 0.2 (0.24) | 1.23 (0.75, 1.99) |  | 0.19 (0.25) | 1.21 (0.74, 2.00) |  | 0.24 (0.24) | 1.27 (0.78, 2.08) |
| $35,000 - $50,000 | 0.19 (0.34) | 1.21 (0.61, 2.36) |  | 0.17 (0.33) | 1.19 (0.61, 2.33) |  | 0.24 (0.33) | 1.27 (0.65, 2.49) |
| $20,000 - $35,000 | 0.45 (0.31) | 1.57 (0.84, 2.94) |  | 0.42 (0.32) | 1.52 (0.81, 2.87) |  | 0.47 (0.32) | 1.61 (0.85, 3.04) |
| < $20,000 | 0.19 (0.29) | 1.21 (0.68, 2.16) |  | 0.14 (0.29) | 1.15 (0.65, 2.06) |  | 0.23 (0.29) | 1.26 (0.7, 2.24) |
| Race/Ethnicity |  |  |  |  |  |  |  |  |
| Non-Hispanic White † |  |  |  |  |  |  |  |  |
| Hispanic | -0.11 (0.43) | 0.9 (0.38, 2.12) |  | -0.09 (0.44) | 0.92 (0.38, 2.20) |  | -0.06 (0.43) | 0.95 (0.39, 2.26) |
| Black | -1.5 (0.46) | 0.22 (0.09, 0.57) |  | -1.49 (0.47) | 0.23 (0.09, 0.58) |  | -1.5 (0.46) | 0.22***(0.09, 0.56) |
| Other | 0.54 (0.43) | 1.71 (0.71, 4.09) |  | 0.55 (0.44) | 1.73 (0.72, 4.2) |  | 0.65 (0.44) | 1.92 (0.79, 4.64) |
| Male | -0.44 (0.25) | 0.65 (0.39, 1.07) |  | -0.4 (0.26) | 0.67 (0.40, 1.13) |  | -0.46 (0.25) | 0.63 (0.38, 1.05) |
| Age (years) | 0 (0.01) | 1 (0.99, 1.01) |  | 0 (0.01) | 1 (0.98, 1.01) |  | 0 (0.01) | 1.00 (0.99, 1.01) |
| Preferred Language: Spanish | 1.11 (0.47) | 3.03*(1.19, 7.74) |  | 1 (0.47) | 2.72*(1.05, 7.01) |  | 1.1 (0.48) | 3.00*(1.15, 7.85) |
| Education |  |  |  |  |  |  |  |  |
| Bachelors or Higher † |  |  |  |  |  |  |  |  |
| Some College | 0.09 (0.21) | 1.09 (0.71, 1.67) |  | 0.07 (0.2) | 1.07 (0.71, 1.6) |  | 0.1 (0.2) | 1.11 (0.74, 1.67) |
| High School | 0.2 (0.24) | 1.22 (0.76, 1.97) |  | 0.18 (0.23) | 1.19 (0.76, 1.89) |  | 0.29 (0.22) | 1.34 (0.85, 2.1) |
| Less than High School | 0.09 (0.35) | 1.09 (0.54, 2.21) |  | 0.16 (0.35) | 1.18 (0.58, 2.37) |  | 0.23 (0.35) | 1.26 (0.62, 2.57) |

† Reference category; * *p*<0.05; ** *p*<0.01; *** *p*<0.001
